# Treatment-resistant idiopathic multicentric Castleman disease with thrombocytopenia, anasarca, fever, reticulin fibrosis, renal dysfunction, and organomegaly managed with Janus kinase inhibitors: A case report

**DOI:** 10.1097/MD.0000000000032200

**Published:** 2022-12-02

**Authors:** Takuya Kakutani, Takahiro Nunokawa, Naofumi Chinen, Yotaro Tamai

**Affiliations:** a Division of Rheumatology, Shonan Kamakura General Hospital, Kanagawa, Japan; b Division of Rheumatology, Tama Nambu Chiiki Hospital, Tokyo, Japan; c Division of Hematology, Shonan Kamakura General Hospital, Kanagawa, Japan.

**Keywords:** anasarca, difficult to treat, interleukin 6, Janus kinase inhibition, TAFRO syndrome

## Abstract

Thrombocytopenia, anasarca, fever, reticulin fibrosis, renal dysfunction, and organomegaly (TAFRO) syndrome are nonmalignant but life-threatening systemic inflammatory disorders. However, many patients are refractory to treatment, resulting in significant morbidity and mortality. Additionally, established treatment options are unavailable. Therefore, we present 2 cases of adults with the iMCD-TAFRO syndrome refractory to initial treatment but responded to Janus kinase (JAK) inhibitors with ruxolitinib. The report reveals that these rare adult cases of the refractory and treatment-resistant iMCD-TAFRO syndrome can be treated using JAK inhibitors.

**Patient concerns:**

Case 1 is a 36-year-old previously healthy male patient who presented with fever and general fatigue for 2 weeks. Case 2 is a 42-year-old previously healthy female patient who presented with fever and general fatigue.

**Diagnosis:**

The diagnosis met the 2015 criteria for TAFRO syndrome, as determined by All Japan TAFRO Syndrome Research Group in the Research Program for Intractable Disease by the Ministry of Health, Labor and Welfare (MHLW) Japan.

**Interventions:**

Treatment with tocilizumab and several immunosuppressants were ineffective. So, we performed ruxolitinib.

**Outcomes:**

Each patient received ruxolitinib, the general condition improved, and CRP levels decreased.

**Lessons:**

These cases showed that ruxolitinib was effective for treatment-resistant/ refractory TAFRO syndrome. Further prospective studies are needed on using ruxolitinib with a small number of cases.

## 1. Introduction

Castleman disease (CD) is a rare cytokine-induced lymphoproliferative and life-threatening disorder,^[1]^ which is classified into unicentric and multicentric CD (MCD) based on the clinical presentation and lymph nodes at the anatomical site. Human herpesvirus 8 (HHV-8) has been confirmed as an etiologic agent in MCD pathogenesis.^[2]^ HHV-8 is found in patients with human immunodeficiency virus (HIV)-associated MCD and those with HIV-negative MCD. Therefore, in patients with MCD of unknown cause, such as those who are HHV-8-negative, the etiology is classified as idiopathic multicentric Castleman disease (iMCD).^[3]^ However, the pathological cell types, dysregulated signaling pathways, and driver cytokines of iMCD remain unknown.^[4]^ Additionally, a poor understanding of its etiology and pathogenesis has contributed to poor patient outcomes. The most severe iMCD subtype is the iMCD thrombocytopenia, anasarca, fever, reticulin fibrosis, renal dysfunction, and organomegaly (TAFRO) syndrome, which involves systemic inflammation.^[5]^ First-line treatment for iMCD syndrome includes glucocorticoids and IL-6 inhibitors; however, approximately 66% of patients with iMCD are IL-6 inhibitor-resistant.^[6]^ Meanwhile, the treatment for recurrent cases has not yet been established. Here, we report 2 rare cases of adult treatment-resistant and refractory iMCD-TAFRO syndromes that responded to Janus kinase (JAK) inhibitors.

### 1.1. Case 1

**Figure 1.**
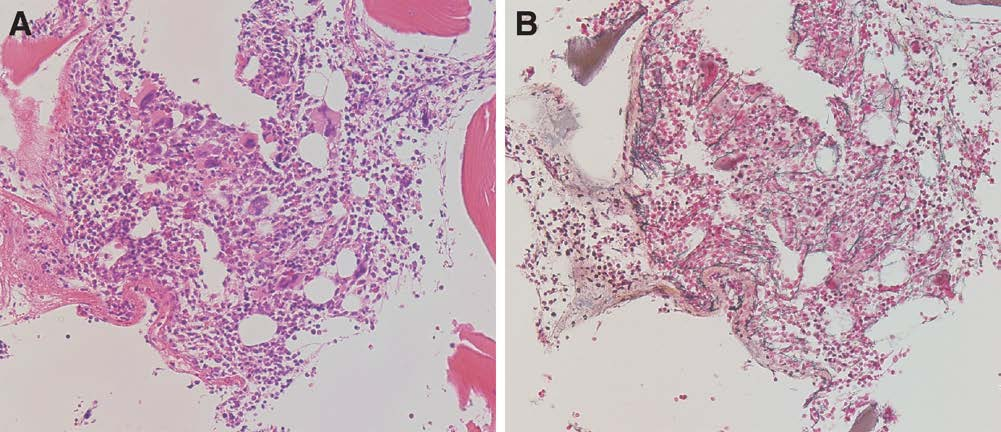
Bone marrow biopsy. (A) Hematoxylin and eosin stain, high power field. (B) Silver impregnation stain, high power field. An increased number of megakaryocytes and reticulin fibrosis were observed.

**Figure 2.**
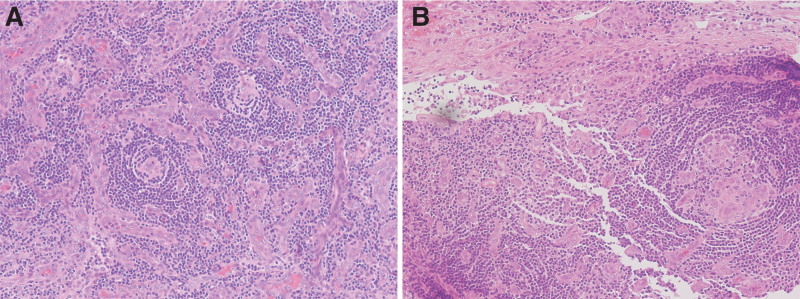
Lymph node biopsy. (A) Hematoxylin and eosin stain, low power field. (B) Hematoxylin and eosin stain, high power field. Severe germinal atrophy and small blood vessel hyperplasia with an enucleated vascular endothelium.

**Figure 3.**
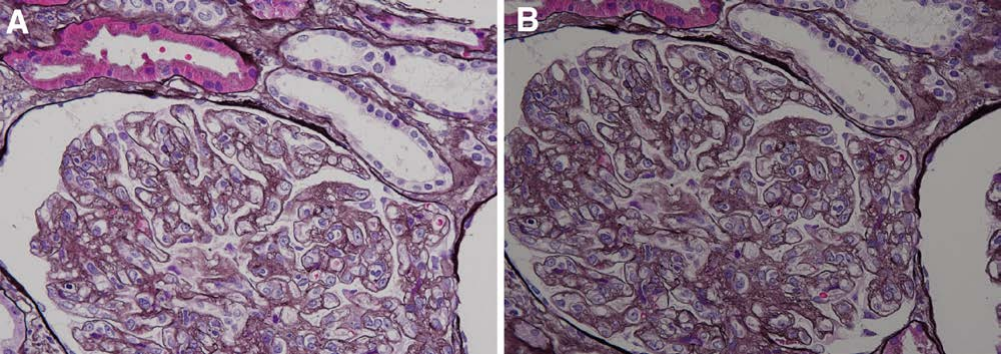
Renal biopsy. (A) Periodic acid-Schiff stain, high power field. (B) Periodic acid-methenamine silver stain, high power field. The glomeruli were lobular, and the endothelial cells were swollen. An increased mesangial substrate was observed.

**Figure 4.**
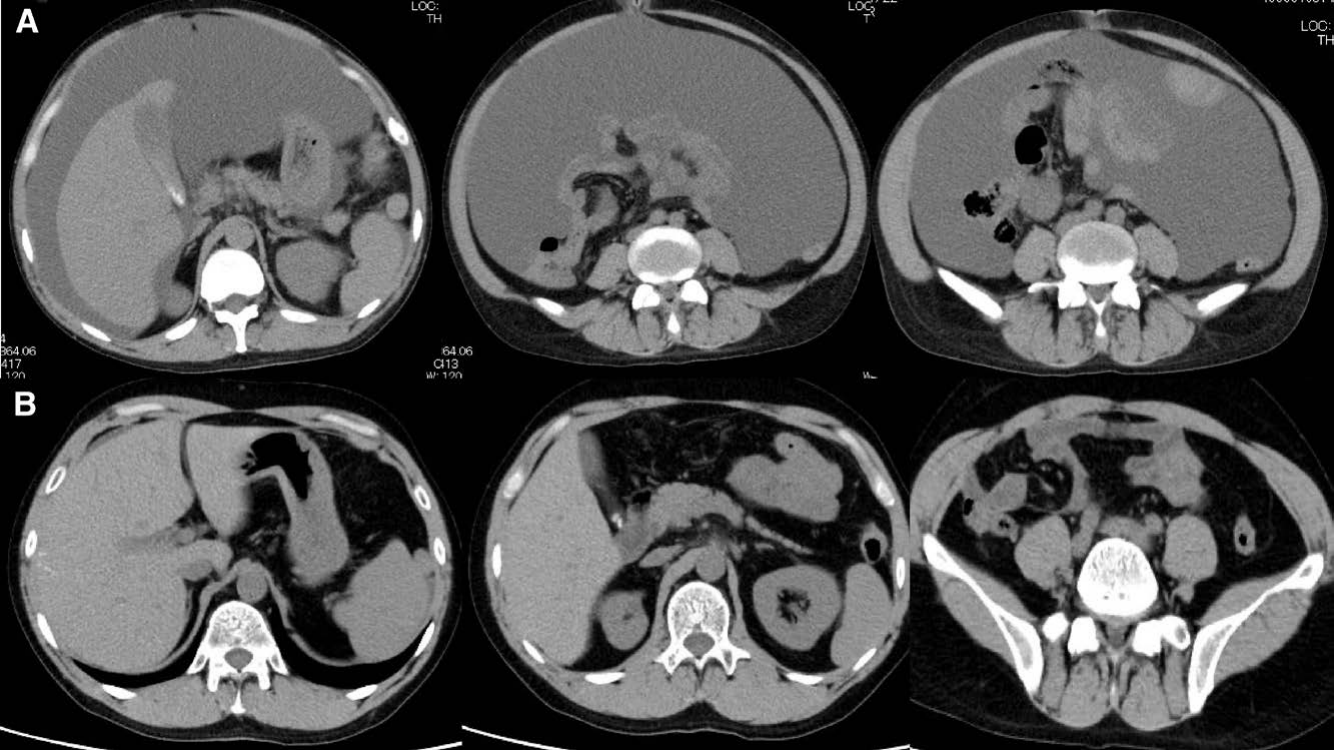
Abdominal computed tomography image. (A) A large number of ascites is observed before ruxolitinib. (B) Resolution of ascites after treatment with ruxolitinib.

**Figure 5.**
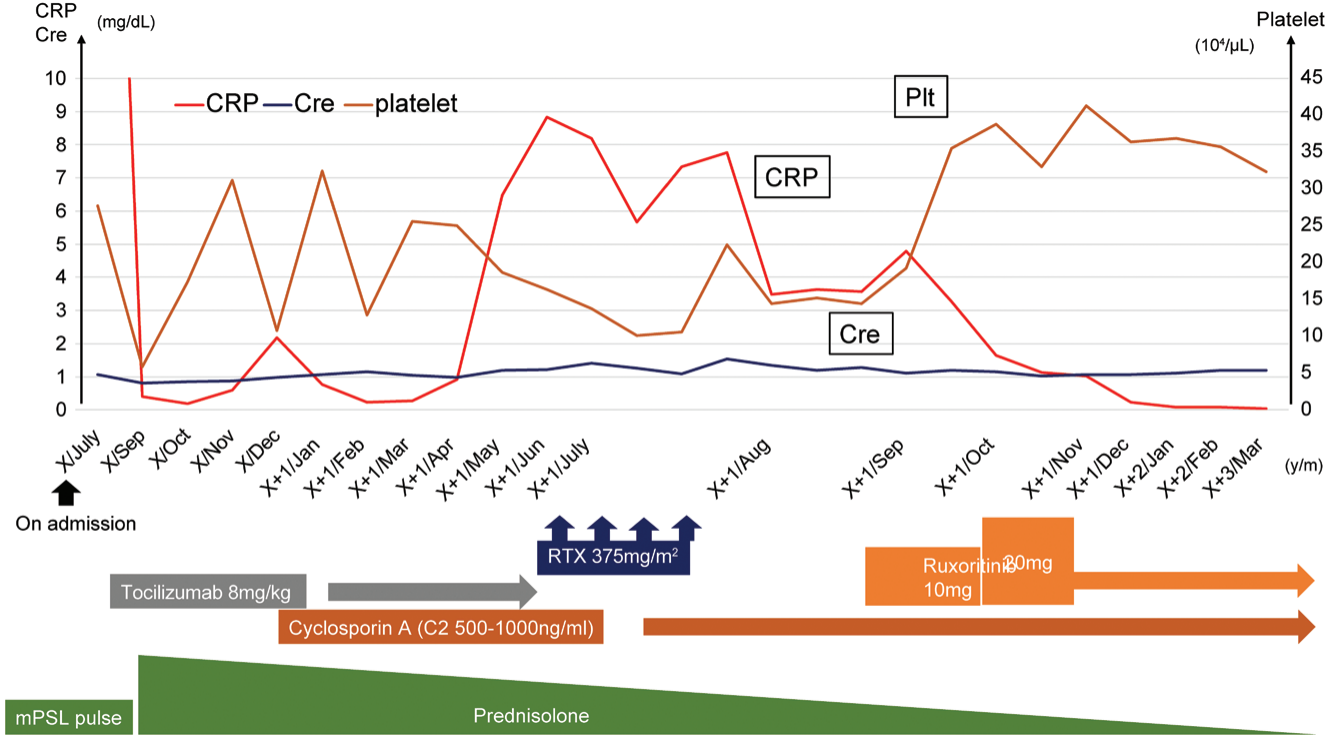
The clinical course after hospitalization. Cre = creatinine, CRP = C-reactive protein, mPSL = methylprednisolone, Plt = platelet, RTX = rituximab.

A 36-year-old previously healthy male patient presented with fever and general fatigue for 2 weeks. He was admitted to our hospital with ambulation difficulties. On admission, his vital signs were as follows: temperature, 38.6°C; blood pressure, 136/74 mm Hg; pulse rate, 116/minutes; respiratory rate, 24/minutes; and SpO_2_, 98% on room air. Heart and lung auscultation results were normal. However, his abdomen was flabby but soft. Small lymph nodes were palpable in the left axilla. Laboratory test results were as follows: complete blood cell counts were normal; C-reactive protein (CRP), 33.6 mg/dL; alkaline phosphatase, 1482 U/L; and lactate dehydrogenase, 182 U/L. Tests for HIV, human T-cell leukemia virus type 1, and HHV-8 were all negative. The soluble interleukin-2 receptor and IL-6 levels were 972 U/mL and 25.6 pg/mL, respectively (Table 1). Although fluid retention was not noted on admission, chest and abdominal computed tomography (CT) examinations showed increased pleural effusion and ascites. Additionally, a liver biopsy was performed because of increased hepatic enzyme levels, and lymphocyte infiltration in the Grisson’s capsule and sinusoids was noted. A bone marrow biopsy was performed since hematological malignancy was suspected, although it did not reveal any hematological disease. However, megakaryocyte hyperplasia and reticulin fibrosis were observed (Fig. 1), and it was MF-2 according to the World Health Organization (WHO) 2016 myelofibrosis classification. A left axillary lymph node biopsy showed CD3-positive lymphocyte proliferation, severe germinal atrophy, and small blood vessel hyperplasia with an enucleated vascular endothelium (Fig. 2). Flow cytometric analysis of lymph node cells revealed a normal kappa/lambda light-chain ratio. The findings met the clinical criteria for TAFRO syndrome and led to the iMCD-TAFRO syndrome diagnosis. Prednisolone (PSL) and tocilizumab (TCZ) at 60 mg/day and 8 mg/kg/week, respectively, were initiated; however, poor treatment response was observed, general malaise persisted, and CRP remained elevated. Since the amount of pleural effusion and ascites increased with poor treatment response, methylprednisolone (mPSL) pulse therapy was initiated post-treatment with mPSL at 80 mg/day. Additionally, the continued administration of TCZ gradually improved the patient’s CRP level and fluid status. However, the PSL dose was gradually reduced while weekly TCZ was continued, and the patient was subsequently discharged with instructions for regular follow-up at the outpatient department. The 13th dose of TCZ was administered on the 10th day following the last dose rather than the 7th day since the fluid retention and CRP levels improved. However, the patient’s fluid-volume status and CRP level worsened, prompting a reversal to weekly TCZ administration beginning with the 14th dose. Although the patient’s general condition was stable, cyclosporine A (CyA) was initiated because elevated CRP and ascites persisted, requiring the drainage of 1500 mL fluid once a month. Furthermore, gradual deterioration of renal function was observed (blood urea nitrogen, 28.9 mg/dL; creatinine [Cre], 1.40 mg/dL) and urinary protein levels (5.15 g/g ▪ Cre). Moreover, the CRP level remained elevated at 8.19 mg/dL. A renal biopsy was performed to determine whether the iMCD-TAFRO syndrome caused renal dysfunction, revealing lobular glomeruli and swollen endothelial cells. The increased number of mesangial substrates was also observed (Fig. 3). The iMCD-TAFRO syndrome progression was considered based on the presence of membranoproliferative glomerulonephritis-like lesions due to iMCD-TAFRO syndrome. Rituximab (RTX) induction therapy was initiated at 375 mg/m^2^ 4 times a week after TCZ was deemed ineffective. After the 4 induction therapy courses, CRP slightly improved to 3.64 mg/dL; however, severe abdominal distension due to ascites persisted, requiring drainage every 2 weeks. His CRP and IL-6 levels (23.4 pg/mL) remained elevated. Hence, the patient was deemed treatment-resistant to TCZ and RTX. Subsequently, ruxolitinib was initiated 13 months after the diagnosis with an initial dose of 10 mg, which was increased to 20 mg. Immediately after initiating ruxolitinib, CRP levels decreased, and fluid retention and renal dysfunction (Cre, 1.07 mg/dL) improved (Fig. 4A and B) (Table 2). After 3 months of oral ruxolitinib treatment, the disease was controlled, and the CRP and renal functions were normalized (Cre, 1.06 mg/dL; urine protein, 0.60 g/g ▪ Cre; CRP, 0.24 mg/dL). The patient is currently on 5, 150, and 20 mg of PSL, CyA, and ruxolitinib, respectively (Fig. 5).

**Table 1:** Laboratory data on admission.

**Hematology**	
WBC	8900/μL
RBC	36810^4^/μL
Hb	10.6 g/dL
Ht	31.9%
Plt	27.710^4^/μL
MCV	86.7 fL
Neut	76%
Lym	14%
Urinalysis	
Protein	(1+)
blood	(–)
RBC	1</HPF
WBC	1-4/HPF
**Biochemistry**	
TP	5.1 g/dL
Alb	1.6 g/dL
T-Bil	1.3 mg/dL
AST	29 U/L
ALT	51 U/L
LDH	182 U/L
ALP	1482 U/L
γGTP	177 U/L
BUN	18 mg/dL
Cre	1.06 mg/dL
Glu	96 mg/dL
CRP	33.6 mg/dL
**Immunology**	
HIV-Ab/Ag	0.8
HHV-8	no detection
ANA	<40
Anti-DNA Ab	<2.0 IU/mL
Anti-SS-A Ab	<1.0 U/mL
C3	147 mg/mL
C4	20 mg/mL
ferritin	585 ng/mL
IL-6	25.6 pg/mL

ALP = alkaline phosphatase, CRP = C-reactive protein, HHV-8 = human herpesvirus 8, IL-6 = interleukin 6, Plt = platelet.

**Table 2:** Laboratory data before and after of ruxolitinib.

	**Pre ruxolitinib (before 1 mo**)	**At the time of ruxolitinib**	**Post ruxoritinib (after 1 mo**)
ALP (U/L)	431	605	348
Creatinine (mg/dL)	1.20	1.20	1.07
Platelet (10^4^/μL)	15.2	35.5	33.0
CRP (mg/dL)	3.64	3.27	1.13
IL-6 (pg/mL)	-	23.4	19.6

ALP = alkaline phosphatase, CRP = C-reactive protein, IL-6 = interleukin 6.

### 1.2. Case 2

**Figure 6.**
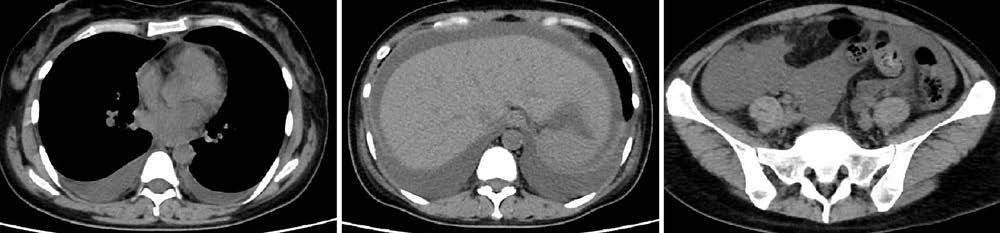
Abdominal computed tomography image on admission. Presence of pleural and ascitic effusion.

A 42-year-old previously healthy female patient presented with fever and general fatigue. Thrombocytopenia, progressive anemia, bilateral leg edema, fever, and renal dysfunction were also noted, and she was transferred to our hospital for further examination. On admission, her vital signs were as follows: temperature, 38.0°C; blood pressure, 140/93 mm Hg; pulse rate, 120/minutes; respiratory rate, 16/minutes; and SpO_2_, 98% on room air. Heart and lung auscultation results were also normal. Abdominal CT showed mild splenomegaly and pleural and ascites effusions suspected of malignant lymphoma; however, no lymphadenopathy was observed (Fig. 6). The CRP level and platelet counts were 26.6 mg/dL and 6.3 × 104/µL, respectively. The HIV, human T-cell leukemia virus type 1, and HHV-8 tests were all negative. On physical examination, she had dry eyes and mouth. The Gam and Schirmer tests were performed, and both were positive.

**Figure 7.**
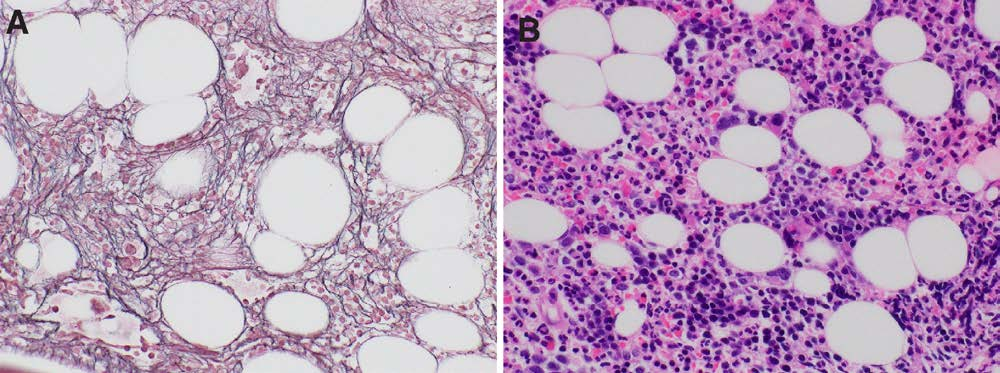
Bone marrow biopsy. (A) Hematoxylin and eosin stain, high power field. (B) Silver impregnation stain, high power field. Increased megakaryocytes and reticulin fibrosis were observed.

Additionally, the patient was positive for anti-SS-A/SS-B antibodies (Table 3). Based on these findings, Sjogren’s syndrome was highly suspected; however, fever, elevated CRP, thrombocytopenia, ascites, bilateral leg edema, and splenomegaly could not be explained, and random skin and bone marrow biopsies were performed to exclude hematological diseases such as intravascular lymphoma. Fever and prolonged CRP elevation levels resulted in significant weakness, and mPSL pulse therapy and CyA were initiated. However, CyA was discontinued on the third day because of adverse events. Bone marrow biopsy showed a mild hyperplastic spinal cord with a few megakaryocytes and large atypical cells infiltrating the intravascular space. Flow cytometric analysis of bone marrow cells revealed a normal kappa/lambda light-chain ratio. Therefore, from the above results, intravascular lymphoma (IVL) was suspected, and cyclophosphamide, hydroxydaunorubicin, oncovin, and prednisone therapy was initiated. The 2 doses also had many inconsistencies with IVL, and reticular fiber hyperplasia was observed when the bone marrow biopsy results were reviewed (Fig. 7). It was MF-2 according to the WHO 2016 myelofibrosis classification. The JAK2 assay result was also negative, considering the possibility of myelofibrosis.

**Table 3:** Laboratory data on admission.

**Hematology**	
WBC	13200/μL
RBC	22710^4^/μL
Hb	6.3 g/dL
Ht	17.9%
Plt	2.510^4^/μL
MCV	78.9 fl
Neut	82%
Lym	9.7%
**Urinalysis**	
Protein	(1+)
blood	(1+)
RBC	1-4/HPF
WBC	1-4/HPF
**Biochemistry**	
TP	6.1 g/dL
Alb	2.5 g/dL
T-Bil	0.8 mg/dL
AST	22 U/L
ALT	13 U/L
LDH	207 U/L
ALP	405 U/L
γGTP	64.9 U/L
BUN	1.79 mg/dL
Cre	1.06 mg/dL
Glu	109 mg/dL
CRP	6.69 mg/dL
**Immunology**	
HIV-Ab/Ag	0.26
HHV-8	no detection
ANA	<40
Anti-DNA Ab	<2.0 IU/ml
Anti-SS-A Ab	>240 U/ml
Anti-SS-B Ab	90.1 U/ml
C3	74 mg/ml
C4	7 mg/ml
ferritin	692 ng/ml
IL-6	4.5 pg/ml

ALP = alkaline phosphatase, CRP = C-reactive protein, HHV-8 = human herpesvirus 8, IL-6 = interleukin 6, Plt = platelet.

**Figure 8.**
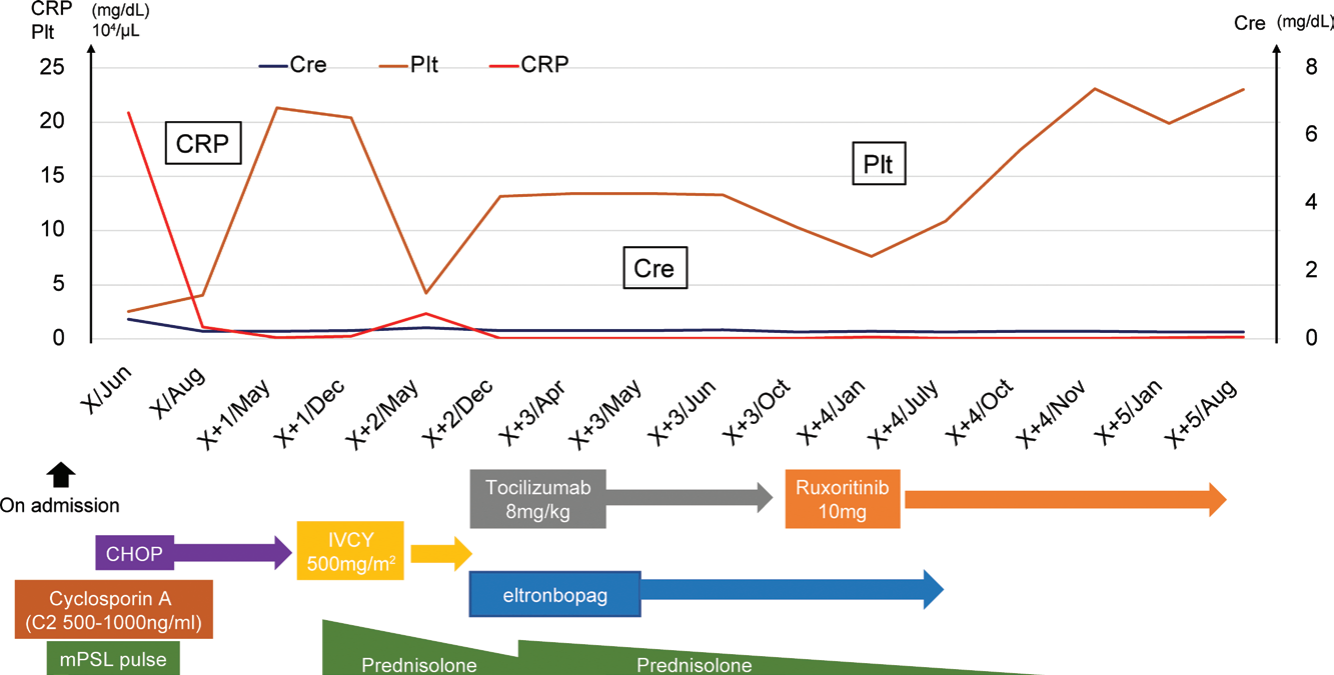
The clinical course after hospitalization. CHOP = cyclophosphamide, hydroxydaunorubicin, oncovin, and prednisone, Cre = creatinine, CRP = C-reactive protein, IVCY = intravenous cyclophosphamide, mPSL = methylprednisolone, Plt = platelet.

Furthermore, the findings met the clinical criteria for TAFRO syndrome based on the results of the bone marrow biopsy and the patient’s clinical course. However, the diagnosis was changed to TAFRO syndrome. As the patient’s condition stabilized, PSL was gradually tapered, and intravenous cyclophosphamide (IVCY) at 500 mg/m^2^/month was concomitantly administered at 5 doses. Conversely, thrombocytopenia recurred 12 months after admission, and 10 mg PSL was restarted. A biopsy was performed because of lymphadenopathy. However, the patient had severe thrombocytopenia, discontinued because she had a poor response to blood transfusion, and was judged high risk. Hence, 8 mg/kg TCZ was initiated 14 months after admission, and eltrombopag was administered. As the patient’s condition improved, the TCZ dosage was reduced to every 3 weeks, and eltrombopag and PSL were discontinued. Sixteen months after admission, the platelet count decreased (7.9 × 10^4^/µL), and the alkaline phosphatase level (410 U/L) increased. Therefore, TCZ- and IVCY-resistant TAFRO syndromes were considered. The platelet count progressed using CyA, IVCY, and TCZ, and the bone marrow fibrosis worsened when the bone marrow was reexamined. We have selected ruxolitinib 10 mg/day, which resulted in rapid improvement in the patient’s blood parameters and general condition (Table 4). The patient’s condition is currently stable, and the disease has been controlled. Finally, PSL was discontinued, and ruxolitinib was maintained at 5 mg/day (Fig. 8).

**Table 4:** Laboratory data before and after of ruxolitinib.

	**Pre ruxolitinib (before 1 mo**)	**At the time of ruxolitinib**	**Post ruxoritinib (after 1 mo**)
ALP (U/L)	317	410	302
Creatinine (mg/dL)	0.9	0.7	0.7
Platelet (10^4^/μL)	9.8	7.9	10.6
CRP (mg/dL)	0.12	0.08	0.01
IL-6 (pg/mL)	4.59	17	-

ALP = alkaline phosphatase, CRP = C-reactive protein, IL-6 = interleukin 6.

In cases 1 and 2, off-label medications were used after receiving approval.

## 2. Discussion

We report 2 recurrent and treatment-resistant TAFRO syndrome cases that were successfully treated with ruxolitinib. TAFRO syndrome, a clinical subtype of the iMCD-TAFRO, is a rare lymphoproliferative disease characterized by systemic inflammation. IL-6 primarily causes systemic inflammation, while vascular endothelial growth factor contributes to its onset. However, renal findings in TAFRO syndrome, which have not been fully established, include glomerular lesions with diffuse global swelling of the endothelium and subendothelial space expansions consistent with the severe glomerular endothelial injury.^[7]^ In case 1, renal pathology was evaluated as the progressed disease, with membranoproliferative glomerulonephritis-like lesions.

Additionally, glucocorticoids and IL-6 inhibitors are the first-line treatment for iMCD-TAFRO.^[8]^ CyA is frequently included in refractory cases or those with inadequate treatment response. RTX^[9]^ and IVCY^[10]^ are also frequently initiated in severe cases with difficult disease control. Although reports on the effectiveness of cyclophosphamide, hydroxydaunorubicin, oncovin, and prednisone therapy, lenalidomide, thalidomide, sirolimus, and siltuximab^[11]^ for the iMCD-TAFRO syndrome exist, no established treatment method is currently available since the disease is rare. The mortality rate was high in cases with poor treatment response. Increased mechanistic target of rapamycin (mTOR) activation has been observed in the lymph nodes of patients with the iMCD-TAFRO syndrome, whereas the iMCD-TAFRO syndrome pathophysiology remains unclear.

In July 2019, sirolimus was used to treat patients with IL-6 inhibitor-resistant iMCD.^[12]^ Clinical trials are currently being conducted with sirolimus.^[13]^ Langan Pai RA et al^[14]^ noted that interferon (IFN)-β stimulation increased mTOR activation in monocytes and T cells of patients with the iMCD-TAFRO syndrome in remission, and type I IFN-induced mTOR activation in patients with the iMCD-TAFRO syndrome. These results indicate mTOR as an inducible cytokine. Type I IFN is a cytokine with 3 major functions. First, they induce cell-specific antimicrobial states in infected and neighboring cells, limiting the pathogens from spreading. Second, they modulate innate immune responses to promote antigen presentation and natural killer cell function while restraining proinflammatory cytokine production. Third, it promotes antigen-specific T- and B-cell responses and immunological memory development. However, type I IFN can also adversely affect autoimmune diseases.^[15]^

Type I IFN has been detected in various systemic inflammatory diseases, and its involvement in systemic lupus erythematosus (SLE) and dermatomyositis (DM) has been investigated.^[16]^ All type I IFN family members, including IFN-α and IFN-β, bind to a signal receptor complex comprising IFN-alpha and beta receptor subunit 1 (AR1) and IFN-AR2, which activates the JAK-signal transducer and activator of transcription signaling pathway.^[17]^ It was shown that IFN-β-induced mTOR activation depends on both JAK and mechanistic target of rapamycin complex 1 (mTORC1) signaling, suggesting that JAK and mTORC1 inhibitors may be effective as therapeutic agents for the iMCD-TAFRO syndrome. The JAK 1/2 inhibitor ruxolitinib is commonly used to treat myelofibrosis.^[18]^ Although the iMCD-TAFRO syndrome pathophysiology has not been identified, type I IFN is involved in its pathogenesis, and IFN-β-induced mTOR activation depends on both JAK and mTORC1 signaling. Therefore, JAK inhibitors might be effective.

Furthermore, a characteristic clinical symptom of the iMCD-TAFRO syndrome is bone marrow fibrosis; the mechanism of myelofibrosis is believed to be the self-proliferation of cytokine-independent hematopoietic cells by JAK 2 activation. Ruxolitinib is a JAK 1/2 inhibitor, which was selected because it is deemed the most effective after considering its involvement in pathogenesis. Moreover, treating severe refractory TAFRO syndrome remains challenging since it is a rare but fatal disease. Two reports of ruxolitinib use in pediatric and adult cases of iMCD-TAFRO syndrome have been reported.^[19,20]^ Therefore, we report 2 rare cases of ruxolitinib use in adults with the iMCD-TAFRO syndrome. However, further prospective studies should confirm the efficacy of JAK inhibitors for the treatment-resistant and refractory iMCD-TAFRO syndromes.

## Acknowledgments

We would like to thank Editage (www.editage.com) for English language editing.

## Author contributions

**Conceptualization:** Takuya Kakutani, Takahiro Nunokawa.

**Data curation:** Yotaro Tamai.

**Investigation:** Takahiro Nunokawa, Naofumi Chinen, Yotaro Tamai.

**Methodology:** Takahiro Nunokawa.

**Project administration:** Takahiro Nunokawa.

**Supervision:** Takahiro Nunokawa.

**Validation:** Takahiro Nunokawa, Naofumi Chinen, Yotaro Tamai.

**Visualization:** Takahiro Nunokawa.

**Writing – original draft:** Takahiro Nunokawa.

**Writing – review & editing:** Takahiro Nunokawa.
